# The Editor's Letter-Box

**Published:** 1915-02-20

**Authors:** 


### THE EDITOR'S LETTER-BOX.

### The American Women's War Hospital.

*To the Editor of* The Hospital.

Medical Superintendent.

*To the Editor of* The Hospital.

Sir,—Apropos of some remarks in the course of this discussion is the following (true) story. It gives some idea of the general ignorance, prejudice, and lack of interest concerning infirmary work.

Not very long ago—in fact, at the time of the South African war—a lady remarked to a friend: "I had to go to the ——— Infirmary the other day to inquire about a soldier's wife, and, do you know, the doctor there was quite a gentleman, just like a doctor in the West End!"

She was not aware that the medical superintendent referred to was well known to her friend.—Believe me, Sir, yours faithfully,

THE "GENTLEMAN" IN QUESTION.

